# Prevalence of TMD and level of chronic pain in a group of Brazilian adolescents

**DOI:** 10.1371/journal.pone.0205874

**Published:** 2019-02-08

**Authors:** Paulo Correia de Melo Júnior, João Marcílio Coelho Netto Lins Aroucha, Manuela Arnaud, Maria Goretti de Souza Lima, Simone Guimarães Farias Gomes, Rosana Ximenes, Aronita Rosenblatt, Arnaldo de França Caldas

**Affiliations:** 1 Postgraduate Program in Dentistry, Department of Pediatric Dentistry, School of Dentistry, University of Pernambuco, Camaragibe, Pernambuco, Brazil; 2 Postgraduate Program in Dentistry, Federal University of Pernambuco, Recife, Pernambuco, Brazil; 3 Department of Prosthesis and Oral Facial Surgery, Federal University of Pernambuco, Recife, Pernambuco, Brazil; 4 Department of Neuropsychiatry, Federal University of Pernambuco, Recife, Pernambuco, Brazil; 5 Department of Pediatric Dentistry, School of Dentistry, University of Pernambuco, Camaragibe, Pernambuco, Brazil; University of Sao Paulo Medical School, BRAZIL

## Abstract

**Aims:**

To determine the prevalence of temporomandibular disorders and associated factors in an adolescent sample from Recife, Brazil.

**Materials and methods:**

A cross-sectional study was conducted with 1342 adolescents aged 10–17 years. The Research Diagnostic Criteria for Temporomandibular Disorders (RDC/TMD) was used by calibrated examiners to evaluate the presence and levels of chronic pain. To evaluate the socioeconomic conditions, the subjects answered the Brazilian Economic Classification Criteria (CCEB) questionnaire. Data were analyzed by means of binary logistic regression in SPSS.

**Results:**

The results showed that 33.2% of the subjects had TMD irrespective of age (p = 0.153) or economic class (p = 0.653). Statistically significant associations were found between TMD and female gender (p = 0.017), headache/migraine in the past six months (p<0.001), chronic pain (p<0.001) and chronic pain level (p<0.001). In the final model, logistic regression showed that the level of chronic pain and the headache/migraine in the past six months were related to the presence of TMD.

**Conclusions:**

The prevalence of TMD was considered high (33.2%) and adolescents with chronic pain and headache in the past six months were more likely to have TMD.

**Clinical relevance:**

The data contribute to the understanding of TMD among adolescents and to the development of preventive measures and polices to identify the dysfunction promptly.

## Introduction

The American Academy of Pediatric Dentistry (AAPD) has recognized that disorders of the temporomandibular joint (TMJ), masticatory muscles and associated structures occasionally occur in infants, children and adolescents. Temporomandibular disorders (TMD) is a collective term for a group of musculoskeletal and neuromuscular conditions that include several clinical signs and symptoms, such as pain, headache, TMJ sounds, TMJ locking and ear pain [1], involving the muscles of mastication, TMJ and associated structures [2].

The prevalence of TMD in adolescents has been reported in recent studies showing a percentage of 9.0% to 48.7%, evaluated by the Research Diagnostic Criteria for Temporomandibular Disorders (RDC/TMD) [3] and the Diagnostic Criteria for Temporomandibular Disorders (DC/TMD) [4], as may be seen in Table 1[5–20]. The RDC/TMD serves as an evidence-based diagnostic and classification system to aid in the rational choice of clinical care for TMD patients around the world [21]. It is based on a series of protocolized clinical procedures and on strict diagnostic criteria applied to the most common types of TMD [22]. Some limitations of this protocol were identified, and some of the items that were questioned in the RDC/TMD were the procedures for diagnosing myofascial pain as well as disc reduction with displacement and the feasibility and practical application of selected palpation sites [4,22]. A revised clinical examination protocol, the DC/TMD, has recently been presented. It appears to be valid for identifying the most common pain-related TMD diagnosis with a higher diagnostic sensitivity and specificity when compared to RDC/TMD [4].

**Table 1:** Prevalence of TMD in adolescents by RDC/TMD and DC/TMD.

Authors	Year	Country	Age (years)	N	Prevalence (%)
Bertoli et al [5]	2018	Brazil	10–14	934	34.9
Graue et al [6]	2016	Norway	12–19	210	11.9
Al-Khotani et al [7]	2016	Saudi Arabia	10–18	456	27.2
Aravena et al [8]	2016	Chile	14–16	186	26.8
Franco-Micheloni et al [9]	2015	Brazil	12–14	1094	30.4
Santis et al [10]	2014	Brazil	6–18	110	20.0
Franco et al [11]	2014	Brazil	12–14	1307	30.4
Pizolato et al [12]	2013	Brazil	8–12	82	48.7
Drabovicz et al [13]	2012	Brazil	18–19	200	35.5
Hirsch, Hoffmann & Türp [14]	2012	Germany	10–17	1011	10.2
Tecco et al [15]	2011	Italy	12–15	390	28.2
Barbosa et al [16]	2011	Brazil	8–14	547	39.1
Moyaho-Bernal et al [17]	2010	Mexico	8–12	235	33.2
Pedras RBN [18]	2010	Brazil	15–20	143	44.1
Wu & Hirsch [19]	2010	German/China	13–18	1058	13.9
Pereira et al [20]	2010	Brazil	12	558	9.0

The influence of socioeconomic factors on different health conditions has been widely recognized. Individuals with higher incomes have greater access to information on health and preventive treatment, which can diminish the likelihood of disease progression [21]. These individuals are also less exposed to risk factors such as precarious housing, nutrient-poor foods [23]. A research demonstrated that the poverty was an important condition in exhibiting myofascial pain and joint problems [21] and a recent study [24] showed significant association between symptoms of temporomandibular joint disorder (TMJD) and poorer oral health-related quality of life (OHRQoL).

The cumulative effect of muscle activities increases the likelihood of presenting painful TMD [25]. Prolonged masticatory muscle pain is likely to become a chronic condition, and continuous pain may eventually produce chronic centrally mediated myalgia [26]. When evaluating adolescents diagnosed with moderate to severe TMD, a higher level of electromyographic activity was found in the masseter and temporal muscles at rest and during chewing [27]. Recent findings have suggested that prepubertal children with high levels of sedentary behavior, low levels of cardiorespiratory fitness and low body fat content may have increased likelihood of various pain conditions [28].

The orofacial pain among children and adolescents, which is also a TMD symptom, is an important public health problem [29] and it should be diagnosed as early as possible since late diagnosis can lead to a more severely compromised state resulting from these pathologies, with relevant consequences [30]. Therefore, assessment of the adolescent population, who are often exposed to possible risk factors, is important to establish the epidemiological pattern of TMD and work at prevention level to avoid the occurrence of the pathology in adulthood [30].

Appropriate care of adolescents with chronic pain requires a great deal of time, energy and affection from their parents [31]. However, due to the lack of proper education or information and prevention policies, these parents often do not understand the risks of future problems developing, with great loss of quality of life [32]. Therefore, this cross-sectional study was designed to evaluate the prevalence of TMD and associated factors in adolescents in the age-range of 10–17 years, according to RDC/TMD, with the purpose of contributing to the understanding of TMD among adolescents and to the future development of preventive and therapeutic measures based on scientific evidence.

## Subjects and methods

The present observational, cross-sectional study was conducted in the city of Recife (Pernambuco/Brazil), in compliance with Resolution 466/12 of the Brazilian National Health Council/Ministry of Health and approved by the Research Ethics Committee (Protocol number 0397.0.172.000–11). The data were collected from of the city of Recife that is divided into two regional offices, north and south, owning 165 public schools with 233.913 students, of these, 87.628 were aged from 10–17 years. The study population consisted of adolescents of both genders enrolled in public schools in 2013; and cluster sampling was carried out covering the regions, in which 20 schools were randomly selected to participate in the study. One school declined to participate because it was undergoing a process of reformation and another because it did not consider Axis II of the RDC suitable for use in adolescents, thus two other schools were included to reach the total of 20.

The inclusion criteria were schoolchildren between the ages of 10 and 17, irrespective of gender or ethnicity, who were regularly enrolled and attending formal school activities at the selected schools that agreed to participate in the study; and adolescents who had their parents’ or guardians’ permission to participate in the research. The exclusion criteria were adolescents with neurological disorders; history of tumor in the head and neck; those who were undergoing continued use (or for less than three days) of anti-inflammatory, analgesic and corticosteroid medications, those unable to understand and/or respond to the RDC/TMD and/or CCEB (Research Instruments); history of rheumatic diseases; pain of odontogenic origin, and primary earache.

Adolescents who decided to participate and their guardians received and signed a term of free and informed consent before filling out the questionnaires. After completing the questionnaire, the adolescents were clinically examined by one of the four examiners who had been previously trained and calibrated for the diagnosis of TMD. The calibration was first performed by two examiners, one with prior experience with the RDC/TMD (gold standard examiner), for 12 hours through the International RDC/TMD Consortium. Two other evaluators (trained examiners) were trained and calibrated by the first two, in addition to watching the RDC/TMD exam training video (available at http://www.rdc-tmdinternational.org). They also performed the manual application of 0.45 and 0.90 kg of pressure on a digital scale prepared exclusively for this purpose.

The presence of TMD and the level of chronic pain were assessed by means of the RDC/TMD, Axis I and II. For the diagnosis of TMD, the axis I was used, which presented the following diagnosis: myofascial pain with or without mouth opening limitation (Group 1-G1); disc displacement with and without reduction, and with or without mouth opening limitation (Group 2-G2); and arthralgia, osteoarthritis and osteoarthrosis (Group 3-G3). The prevalence of TMD was calculated by the number of subjects who had at least one positive diagnosis in one of the groups. The level of chronic pain was evaluated by means of Axis II.

The socioeconomic conditions were measured by the Brazilian Economic Classification Criteria (CCEB). The CCEB was developed by the Brazilian Association of Research Companies [33] for population classification into groups according to economic class. This classification, based on the possession of goods and not on family income, has scores varying from zero (the poorest) to 46 (the richest).

The scores were transformed into social class categories. Scores from 0 to 7 correspond to class E, 8 to 13 (class D), 14 to 22 (class C), 23 to 34 (class B), 35 to 46 (class A). In 2013, the Brazilian Association of Research Companies changed this categorization. Thus, at present the classification is Class A1 and A2 (high socioeconomic level), B1 and B2 (medium-high socioeconomic level), C1 and C2 (medium-low socioeconomic level) and D-E Class (as a single class-poor socioeconomic level).

The clinical examination, according to the orientation of Axis I of the Research Diagnostic Criteria for Temporomandibular Disorders, was then performed under natural light and consisted of an extraoral and intraoral examination of the teeth and bite, palpation of the temporalis, masseter, digastric and medial pterygoid muscles, palpation of the temporomandibular joint and an analysis of jaw movement. The participant, seated in a chair, was instructed to close his/her mouth until maximum intercuspidation in centric occlusion. The participant was previously trained to perform this procedure and then instructed to maintain his/her usual bite with maximum clenching to determine the type of occlusion.

Headaches were assessed by means of question #18 of the RDC/TMD Axis II history questionnaire (“During the last six months have you had a problem with headaches or migraines?”) [3]. The degree of chronic TMD pain was also done by RDC/TMD Axis II through the chronic pain protocol evaluated, in which pain-related questions received points, and the sum of these points reported the degree of disability ranging from absence of chronic pain in the last six months (Grade 0) to severe limitation (Grade IV).

The Kolmogorov-Smirnov Z test was used to determine the data distribution (normal or non-normal). The data were first evaluated to obtain their percentages and distributions, and then the associated factors were identified, observing odds ratios (OR) and confidence intervals of 95% (95% CI). Continuous variables were analyzed by using the Chi squared test.

A binary multivariate logistic regression model was constructed, in which only the variables that had a p-value ≤ 0.20 in the bivariate analysis were taken into account. The logistic regression model allowed statistical evaluation of the behavior of a variable, to verify whether the presence of a risk factor increased the probability of a given outcome by a specific percentage. In the analysis, the dependent variable was analyzed, dichotomized as follows: 0 = no signs and/or symptom of TMD, 1 = at least one clinical sign and/or symptom of TMD. The adjustment of the model was evaluated with the Hosmer-Lemeshow test that is frequently used in risk prediction models. In the multivariate analysis, the variables were introduced into the model as dummy variables. All statistical tests were carried out using the Statistical Package for Social Sciences (SPSS) version 23.0.

## Results

**Fig 1:**
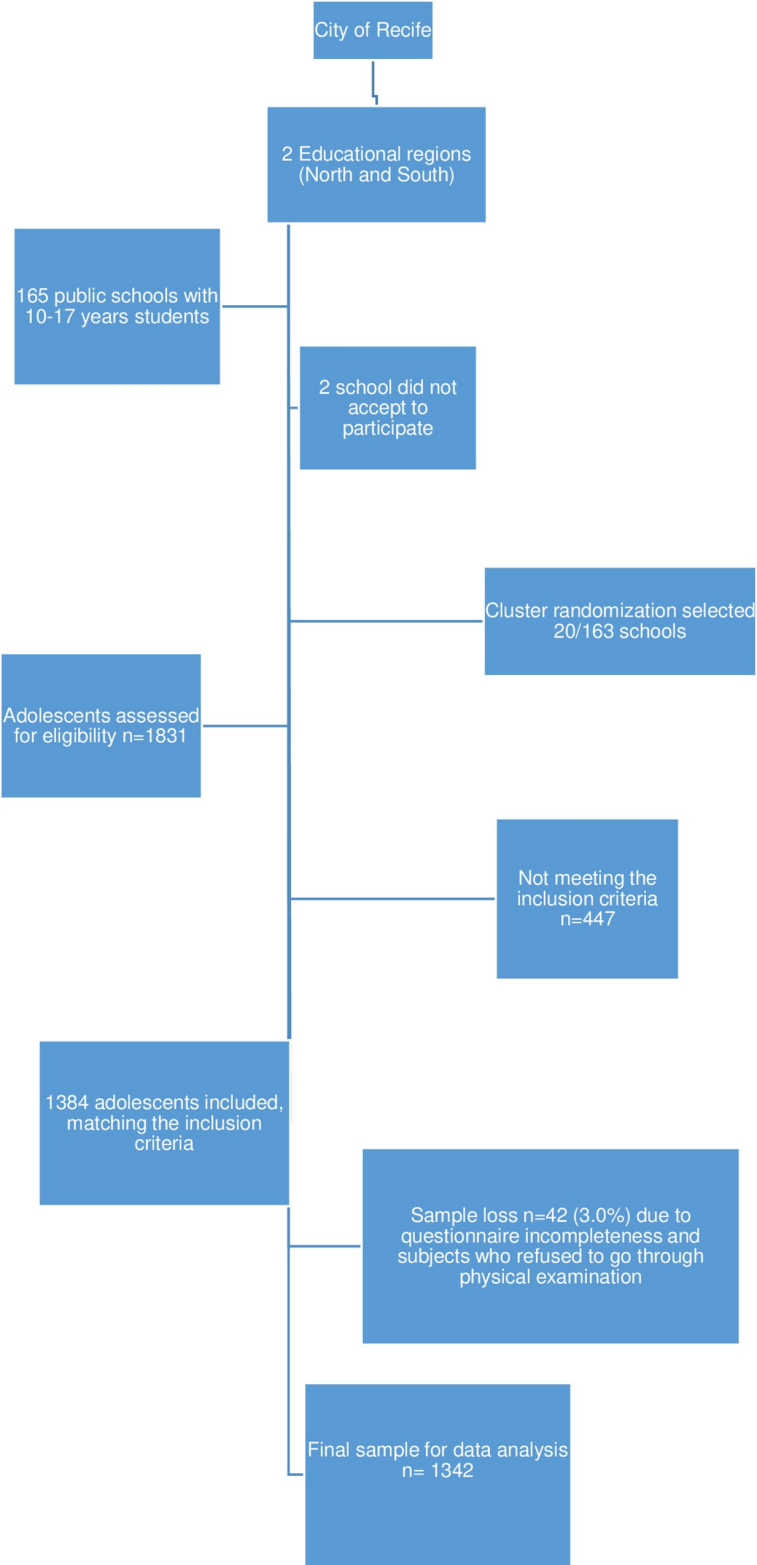
Recruitment, enrollment, randomization, withdrawal and completion of subjects.

The sample size was calculated based on the population of students enrolled in the State Educational System in Recife in the target age-range of search with a 95% confidence interval, a proportion of 0.331 (estimated prevalence of TMD), and the precision was fixed at 0.03, which resulted in a sample size of 1.519 adolescents after a cluster effect of 1.2 and with an additional 20% to cover possible sample loss. All the students were considered pre-eligible (1831 adolescents) and were then assessed according to inclusion and exclusion criteria. After the inclusion criteria, 447 were excluded and 42 were lost (Fig 1). The intra- and inter-examiner reliability levels varied from 0.92 to 0.96 analyzed by Cohen kappa statistics.

The sample consisted of 1342 individuals of whom 68.7% were females; and 60% belonged to medium-low socioeconomic level (class C). The prevalence of TMD in the studied sample was 33.2% with a peak at the age of 12. In the last six months, 70.9% of the adolescents had headache/migraine with a little over one third of them associated with TMD (36.4%). Relative to chronic pain, this was shown in 27.9% of subjects, and in 47.9% of them pain was associated with TMD, 24.6% with low disability (low and high intensity) and 3.2% with high disability (moderately and severely limiting) (Table 2). Joint dysfunction was the most common TMD diagnosis (38.9%), followed by disc displacement (30.3%) and myofascial pain (11.5%) (Table 3).

**Table 2:** Distribution and bivariate analysis of participants regarding TMD according to gender, age, economic class, headache in the past six months and presence and degree of chronic pain.

Variables		TMD		Total	p-value
		Yes (%)	No (%)	N (%)	
**Gender**	Male	120 (28.6)	300 (71.4)	420 (31.3)	0.017* (a)
	Female	325 (35.2)	597 (64.8)	922 (68.7)	
**CCEB**	A + B	155 (32.7)	319 (67.3)	474 (35.3)	0.653 (a)
	C	273 (33.9)	532 (66.1)	805 (60.0)	
	D+E	18 (28.6)	45 (71.4)	63 (4.7)	
**Age (years)**	10–14	286 (34.6)	540 (65.4)	826 (61.5)	0.153 (a)
	15–17	159 (30.8)	357 (69.2)	516 (38.5)	
**Headache in the past six month**	Yes	347 (36.4)	605 (63.6)	952 (70.9)	<0.001* (a)
	No	98 (25.1)	292 (74.9)	390 (29.1)	
**Chronic Pain**	Yes	179 (47.9)	195 (52.1)	374 (27.9)	<0.001* (a)
	No	266 (27.5)	702 (72.5)	968 (72.1)	
**Degree of chronic pain**	No pain	266 (27.5)	702 (72.5)	968 (72.1)	<0.001* (a)
	Low intensity	49 (39.5)	75 (60.5)	124 (9.2)	
	High intensity	99 (7.4)	107 (8.0)	206 (15.4)	
	Moderately limiting	27 (2.0)	11 (0.8)	38 (2.8)	
	Severely limiting	4 (0.3)	2 (0.1)	6 (0.4)	

Chi-square test

*statistically significant

**Table 3:** Absolute and relative numbers of individuals according to their TMD diagnosis, based on RDC/TMD.

TMD diagnosis	n	%
**Group 1: myofascial pain**	51	11.5
**Group 2: disc displacement**	135	30.3
**Group 3: joint dysfunction**	173	38.9
**Group 1 + group 2**	09	2.0
**Group 1 + group 3**	46	10.3
**Group 2 + group 3**	24	5.4
**Group 1+2+3**	07	1.6
**Total**	445	100

We observed that the gender showed statistically significant association with TMD (p = 0.017) and so did headache in the past six months (p<0,001); chronic pain (p<0.001); and degree of chronic pain (p<0.001), whereas no statistically significant associations were found between TMD and age (p = 0.153); and economic class (p = 0.653) (Table 2). Although the independent variable economic class presented a p-value above 0.2, it was also taken into the logistic regression analysis to verify whether it was a confounding variable or whether it functioned as an intervening variable. We found that this variable did not present any of these characteristics. The final multivariate logistic regression model is shown in Table 4. This final model consisted of two principal effects. Chronic pain at almost all levels (exception for the level 4) and headache in the past six months contributes to the presence of TMD.

**Table 4:** Final multivariate logistic regression model.

	B	S.E.	Wald	df	Sig.	Exp(B)	95.0% CI. for EXP(B)	
							Lower	Upper
**Chronic Pain Grade 0**			47.028	3	<0.001			
**Chronic Pain Grade 1**	1.755	0.367	22.810	1	<0.001	5.784	2.815	11.886
**Chronic Pain Grade 2**	1.268	0.403	9.900	1	0.002	3.552	1.613	7.823
**Chronic Pain Grade 3**	0.923	0.385	5.760	1	0.016	2.517	1.184	5.348
**Headache in the past 6 months**	0.343	0.139	6.081	1	0.014	1.409	1.073	1.851
**Constant**	-0.898	0.358	6.302	1	0.012	0.407		

## Discussion

This was a population-based epidemiological study that presented the prevalence of TMD-diagnoses according to the RDC/TMD classification among adolescents aged 10 to 17 years. Epidemiological studies are useful for the management of healthcare services by allowing the profile of a given population to be determined and helping to establish public policies with the aim of controlling and eradicating adverse health conditions [21]. The different prevalence rates described for TMD in the literature may be explained by the use of different diagnostic tools for TMD, absence of clinical examinations and self-reported TMD-pain, signs and symptoms [7, 25]. The RDC/TMD are the most important diagnostic tools, properly translated into Portuguese [34] and other languages, showing good reliability in children and adolescents [35], in addition to being adapted, validated, and extensively used since 1992 [3]. Although there is a new version of the RDC, DC/TMD, this new method of diagnosis has not yet been validated for Brazil and there are still many published articles that allow an adequate comparison with the findings obtained with the former version.

In the present study the prevalence of TMD (33.2%) was determined on the basis of any TMD subtype in Axis I of the RDC/TMD in a sample composed of adolescents aged 10 to 17 years; it was a little higher than values shown in previous literature reports [7, 9, 15] and similar to those shown by others [17]. This could also be attributed to at least two additional factors. First, the age range studied in the present study, not only one age group, also made it difficult to compare their outcomes with those of other studies. Moreover, the adolescents in the present study were diagnosed with TMD irrespective of the type. These results showed that TMD evaluation should be a recommended part of the routine examination. Many adults with TMD pain have reported that their condition began during adolescence [36]. Individuals who developed TMD pain in adolescence may have had an underlying vulnerability to experiencing pain that was not restricted to the orofacial region [37].

The presence of reproductive hormones seemed to increase the risk of developing pain during the time when girls go through puberty [38]. However, no evidence has been found up to the present time indicating how sex hormones could affect sensory processing in the trigeminal system, especially during adolescence [9, 39] or in association with the menarche [11]. In our study, we found statistically significant association between gender and TMD, which was in disagreement with findings described in previous studies [7–9, 12, 17, 25, 40], but there are other studies that have shown significant association between female gender and TMD, with females being the most affected [6, 14–16, 37, 41, 42]. On the other hand, our results must be analyzed with caution, since there was an unequal proportion between girls and boys evaluated; twice as many girls volunteered to participate in the study.

The prevalence of TMD increased from childhood up to young adulthood [15, 43]. In this study, the prevalence of TMD was found to be higher in early adolescence (61.5%) than in the late stage (38.5%). However, within the period of adolescence there was also a tendency for TMD to increase. Others studies [6, 44] reported that TMD started to increase at the age of 12 and peaked at the age of 16. In our findings, TMD had the highest peak at the age of 12, which could be explained by the presence of reproductive hormones increasing the risk of pain developing during the time of puberty in girls [38]. The bivariate analysis showed no association (p = 0.153) between categorized age and TMD.

Several health problems may be associated with economic class; at present there is no evidence supporting a relationship between economic class and TMD. The majority of adolescents in our study were classified as Class C (60.0%). The results of our study showed no statistical association between socioeconomic conditions and TMD (p = 0.653). However, in the literature there were results in agreement with our study [9] and others in disagreement [16, 24], probably because of the difference in the diagnostic criteria and age groups.

Headaches are the most prevalent neurological disorders and one of the most common symptoms reported in general practice. The percentage of the adult population with an active headache disorder is 46% for headache in general, in children/adolescents rates of up to 69.5% have been reported [40]. In the WHO´s ranking of causes of disability, this would rank headache disorders among the 10 most disabling conditions for the two genders; and the five most disabling for women. Headache is commonly associated with TMD among children and adolescents [11, 40, 45, 46]. Its presence in adolescents may result in low achievement in school, difficulty in social relationships; moreover, difficulty with eating can cause even more pain, and influence their biological functions, loss of quality of life, suffering and disability. It has also been speculated that a combination of developmental and hormonal changes would be responsible for increasing headache in girls after menarche [47], but this could also not be confirmed [11].

Headache makes pain parameters more intense and frequent, complicating dysfunctional diseases both in the diagnostic and treatment phases [48]. In our findings, 70.9% of the adolescents had headache/migraine, and in a quarter of them it was associated with TMD (25.9%) in the past six months (p<0.001). There were significant statistical association between headache in the past six months and TMD, this was in agreement with previous studies [7, 9, 11, 17, 40]. Signs and symptoms of TMD occurred more often in adolescents with headache in comparison with those who were headache-free [49]. This could be explained by the fact that headache determines an increased central sensitization to pain and an exacerbation of pain symptoms in the craniocervical-mandibular joint [50].

There are two important aspects of chronic pain in children and adolescents: the delay in referring these patients to a pediatric pain specialist, and the failure to recognize psychological disorders as an important comorbid condition in chronic pain [51]. Often, lack of an identifiable etiology along with the complex biopsychosocial nature of this condition leads to a lengthy diagnostic odyssey and delayed treatment that exacerbates the existing problem [52].

This populational based Brazilian epidemiological study assessed the degree of chronic TMD pain by means of the RDC/TMD Axis II among adolescents aged 10 to 17 years. Our findings showed that in 27.9% of adolescents there were significant associations between presence of chronic pain and TMD, among whom 24.6% had low disability (low and high intensity without limitation of the function) and 3.2% had high disability (some degree of limitation of the function) (p<0.001). Previous findings have shown association between presence of chronic pain and TMD, in agreement with our findings [7, 9]. Logistic regression showed that the degree of chronic pain and the headache contributed to the presence of TMD. The fact that most adolescents did not have chronic pain (72.1%) could be because the orofacial muscles of young individuals have higher physiological adaptive ability during growth and development.

Some studies have suggested that individuals who reported pain and other common symptoms in childhood are at an increased risk for having pain in adulthood [53–56]. Patients with childhood chronic pain had 3 times more chance to have fibromyalgia, according to the American College of Rheumatology (ACR) survey criteria, in contrast with those who denied chronic pain in their youth. Also consistent with fibromyalgia, or more broadly, the centralized pain phenotype, patients reporting childhood chronic pain had higher levels of anxiety symptoms and slightly worse functional status [57, 58].

The strengths of our study included: a large and representative adolescent study population; the methodology for assessing by RDC/TMD, Axis I and II; the sample size and sampling process were representative of the age group, with results demonstrating a high prevalence. On the other hand, our sample was comprised only adolescents enrolled in the public education system, for this reason, although the sample size and the sampling process was considered very adequate, we could not extrapolate our results to the entire population of adolescents in the municipality.

## Conclusions

- The prevalence of TMD among adolescents was high irrespective of age or economic class;
- The gender, headache/migraine, chronic pain and level of chronic pain had a statistically significant association with TMD;
- The level of chronic pain and the headache/migraine in the past six months contributed to the presence of TMD.

## Supporting information

S1 File Data set from the study. (SAV)
